# Physicians’ ethical concerns about artificial intelligence in medicine: a qualitative study: *“The final decision should rest with a human”*

**DOI:** 10.3389/fpubh.2024.1428396

**Published:** 2024-11-27

**Authors:** Fatma Kahraman, Aysenur Aktas, Serra Bayrakceken, Tuna Çakar, Hande Serim Tarcan, Bugrahan Bayram, Berk Durak, Yesim Isil Ulman

**Affiliations:** ^1^Acibadem University, Departmant of Psychology, Istanbul, Türkiye; ^2^Acibadem University, School of Medicine, Istanbul, Türkiye; ^3^MEF University, Department of Computer Engineering, Istanbul, Türkiye; ^4^Acibadem University, Biomedical Engineering Department, Istanbul, Türkiye; ^5^Acibadem University School of Medicine, History of Medicine and Ethics Department, Istanbul, Türkiye

**Keywords:** artificial intelligence, medicine, healthcare, ethics, decision-making, qualitative research

## Abstract

**Background/aim:**

Artificial Intelligence (AI) is the capability of computational systems to perform tasks that require human-like cognitive functions, such as reasoning, learning, and decision-making. Unlike human intelligence, AI does not involve sentience or consciousness but focuses on data processing, pattern recognition, and prediction through algorithms and learned experiences. In healthcare including neuroscience, AI is valuable for improving prevention, diagnosis, prognosis, and surveillance.

**Methods:**

This qualitative study aimed to investigate the acceptability of AI in Medicine (AIIM) and to elucidate any technical and scientific, as well as social and ethical issues involved. Twenty-five doctors from various specialties were carefully interviewed regarding their views, experience, knowledge, and attitude toward AI in healthcare.

**Results:**

Content analysis confirmed the key ethical principles involved: confidentiality, beneficence, and non-maleficence. Honesty was the least invoked principle. A thematic analysis established four salient topic areas, i.e., *advantages*, *risks*, *restrictions,* and *precautions*. Alongside the advantages, there were many limitations and risks. The study revealed a perceived need for precautions to be embedded in healthcare policies to counter the risks discussed. These precautions need to be multi-dimensional.

**Conclusion:**

The authors conclude that AI should be rationally guided, function transparently, and produce impartial results. It should assist human healthcare professionals collaboratively. This kind of AI will permit fairer, more innovative healthcare which benefits patients and society whilst preserving human dignity. It can foster accuracy and precision in medical practice and reduce the workload by assisting physicians during clinical tasks. AIIM that functions transparently and respects the public interest can be an inspiring scientific innovation for humanity.

## Introduction

Artificial Intelligence (AI) is upon us, and it is transforming our lives and professions. The applications of AI in the health domain (1) have already provided diverse benefits such as facilitating diagnosis (2) and disease classification processes (3), improving health and drug development research helping to expand public health interventions and supervision, enabling the development of personalized medicine (4), as well as calculating risks and costs in various aspects (5). The reliability and validity of AI applications, the confidentiality of the information these applications contain, the risk of them spreading to non-medical environments, the possibility of increasing inequalities in access to health care, and the possibility of disqualification of health workers in clinical studies as in clinical neuroscience whose nature has changed with these applications can be counted among the problems posed by AI applications (6). This situation requires that we tackle the issue comprehensively, figure out the values and duties of the parties, perform an analysis buttressed by ethical principles, in order to reach an ethical stance (7). As recommended by Jackson et al. (8) AI-powered systems are increasingly transforming society and health, and therefore they need to be managed within an ethical framework.

In this respect, UNESCO, through its 2021 “Recommendation on the Ethics of Artificial Intelligence” signed by 193 member states, stated that the ethics of AI should be structured to protect and promote human rights and human dignity, by giving a strong emphasis to the respect for the rule of law in the digital world (9). This inclusive document strongly recommends that the use of AI technologies should be guided by both sound scientific research and ethical analysis and evaluation. In view of the ethical principles of AI implementations, the UNESCO Recommendation attaches importance to the respect of non-maleficence and beneficence, and to the preservation of proportionality in the implementation of methods, measures and risk assessment of AI systems; it emphasizes the need to ensure the safety and security of humans, the environment and the ecosystem through AI applications; to promote social justice and to safeguard fairness and non-discrimination in compliance with international law; to avoid discriminatory and biased applications, and to facilitate equitable access to technology; to respect the right to privacy by protecting human dignity, human autonomy, and human agency through AI systems while they collect, use, share and delete data in ways consistent with international law; and to assure transparency, explainability, accountability and responsibility in the life cycle of AI systems (10).

The Council of Europe (COE) specifies a series of steps necessary to ensure that human rights are guaranteed; these include transparency, independent oversight, non-discrimination and equality, data protection and privacy, and promotion of AI literacy (11). In a similar fashion, the COE recommends understanding the functions of systems that employ automated decision-making, advises making informed decisions in the use of such systems, draws attention to the benefits of utilizing algorithmic systems, and emphasizes the need to minimize the exposure to risks that may stem from using such systems—all this to manage the human rights impacts of algorithmic systems (12). In this sense, the EU AI Act which is the first regulation on AI was adopted by European Parliament in March 2024. This regulation is quite significant to guide the user regarding safety, reliability, transparency and explainability of AI-powered systems (9).

In the last 30 years, AI has shifted from knowledge-based to data-driven algorithms (13). Computerized AI systems exhibit human-like cognition and intelligence and can accomplish tasks that require intelligence, such as altering functions according to perceived environmental alterations (14). Currently, extensive research on AI is being undertaken in various areas, including healthcare, and significant advances in knowledge have been made (15). Siau and Wang (16) argued that the vast socioeconomic benefits of applying AI to tasks such as facial recognition, medical diagnosis and autonomous vehicle driving may be achieved only if AI is programmed ethically.

In this study, AI is understood as the deliberate use of any algorithm implementing a rule-based system, machine, or deep learning to solve a problem (17). Natural Language Processing (NLP), robotics, computer vision, and Brain-Computer Interface (BCI) are directly related domains. It is widely expected that AI will offer many advantages in healthcare, including improvement in prognosis, smarter management and automation of radiological and histopathological diagnostics, more accurate diagnoses, the ability to handle huge amounts of omics data in a very short time and with more precision for the benefit of patients. This will be transformative for medicine and for physician-patient interactions (17). AI may even diagnostically outperform human physicians in fields where vast data sets are available for its training, namely oncology, cardiology, gastroenterology, ophthalmology, clinical neuroscience, and surgery (18).

Amisha et al. (19) carefully examined the use of AI-powered systems in family medicine. Liu et al. (20) illustrated the advantages of AI-based technology for the benefit of human health and safety, in medical diagnosis, medical treatment, medical management and education, drug production, as well as in the coronavirus research. These have provided the initial motivation for our current study and for the choice of a qualitative methodology to pursue this aim.

As a matter of fact, in a qualitative study exploring the awareness and knowledge of radiographs and radiologists on AI-based technologies, the respondents regarded this innovative technology as helpful to counter workforce shortages, but these respondents exhibited varying views regarding AI as an opportunity to take a more defensive or skeptical stance. That study already postulates the existence of a tension between AI and human behavior (21). Similarly, a thematic analysis of 24 interviews questioning the underlying opinions and attitudes of physicians regarding the implementation of computerized clinical decision support systems highlighted that physicians are concerned not only with the technical and ethical aspects of their job, but also with its existential and social values, which make them perceive their work as meaningful. They are doubtful whether a fully automated system would fulfill the uniqueness of the medical profession (22). Another qualitative study with participants from diverse backgrounds inquired the perceptions of AI in healthcare; it concluded that although the participants welcomed the production of high-quality data via artificially intelligent systems in healthcare, they questioned the issue of the responsibility in AI-powered systems, which might eventually menace the beneficence of the patients (23).

This qualitative study aims to better understand the role, from the perspective of physicians in Turkey, of AI-based systems in the context of the medical field, and it tries to elucidate the ethical challenges posed by AI in Medicine (AIIM). The anticipated benefits go alongside significant concerns from patients, the public and healthcare professionals about the risks and opportunities (24). The first task is to set the limits of what AI may or may not do, through regulations, standards, and guidelines. The “Ethics Guidelines for Trustworthy AI” produced by the High-Level Expert Group on AI is an example that sets out a framework in the hope of achieving a trustworthy AI (25). These limits prevent AI from being overestimated (26). Regarding the ethical aspects of AI implementation in healthcare, Vayena et al. (27) called for reciprocal trust, data protection, minimization of bias, and transparency. These requirements may be achieved through regulation, and it is therefore important to take into account the points of view of the individuals who work on the ground in order to grasp the scope of the issue, and to consequently shape healthcare policies in accordance with the properties of emerging technologies.

## Methods

This study was conducted using a single-interview-per-participant qualitative design. The researchers utilized a bespoke interview format and a demographic questionnaire. Questions were generated from the literature and expert advice. Ethical approval was granted by the University Ethics Committee on 28.02.2019 by decree 2019/4–31. Informed consent was obtained from each participant prior to interview.

Participants from different fields of expertise were selected based on purposive sampling. The demographic information obtained related to the physicians’ specialty, the length of service as a physician, the time they practiced as a specialist, and whether they received previous healthcare ethical training. Twenty-five physicians employed through a university were interviewed face-to-face; the interviews were recorded (voice only) with their consent. Purposive sampling utilizing demographic characteristics and survey answers identified candidates for later semi-structured interviews. The data collection process was stopped when data saturation was reached. Table 1 provides details of the participants.

**Table 1:** Demographic details of participants.

	Years	*n*	%
Age	35–44	5	20
	45–54	11	44
	≥55	9	36
Length of service as a doctor	5–14	1	4
	15–24	10	40
	25–34	10	40
	≥35	4	16
Length of time working as a specialist	5–9	1	4
	10–19	13	52
	20–29	8	32
	≥30	3	12
Ethics lessons taken during medical education	Yes	18	72
	No	7	28

Specialist fields of participants: Radiology, Cardiovascular Surgery, General Surgery, Clinical Chemistry, Ophthalmology, Cardiology, Chest Diseases, Dermatology, General Practice, Gastroenterology, Genetics, Hematology, Medical Education, Medical Microbiology, Pathology, Pediatric Allergology, Pediatrics, Physiotherapy and Rehabilitation.

The interview covered knowledge of AI, use of AIIM, sources consulted, professional experience of AI, advantages and disadvantages of AI plus support for its use, how AI is used by physicians, healthcare professionals and patients, effects on healthcare, ethical values involved in using and developing healthcare AI, and willingness to recommend AI-facilitated diagnosis and management to a relative.

### Analysis

Content and thematic analyses were deemed the most appropriate analytical research methods to achieve the study aims. Specifically, participants’ knowledge and experience were investigated alongside the ethical principles related to AIIM. The thematic analysis aimed to uncover patterns in the physicians’ general interview answers. These patterns were categorized in main and sub-themes as well as the basic problems and subproblems were identified in the original data (28). The data analysis was performed with MAXQDA. An open-coded map allowed themes to be defined; the themes were then finalized based on the views of three different specialist researchers.

## Results

This section will first present the descriptive findings from the content analysis, followed by those from the thematic analysis.

### Conclusions from content analysis

The first step of the content analysis was to evaluate the participants’ prior knowledge of AIIM, the origin of this knowledge, and any direct experience they may have of it. The participants that possessed prior experience of AIIM represented 20% (*n* = 5) of the total. The majority of respondents lacked direct experience of AIIM, but 68% (*n* = 17) claimed some degree of knowledge. The main sources for this knowledge were the Internet or the media (*n* = 11), books or articles (*n* = 10), and conferences or seminars (*n* = 7).

**Figure 1:**
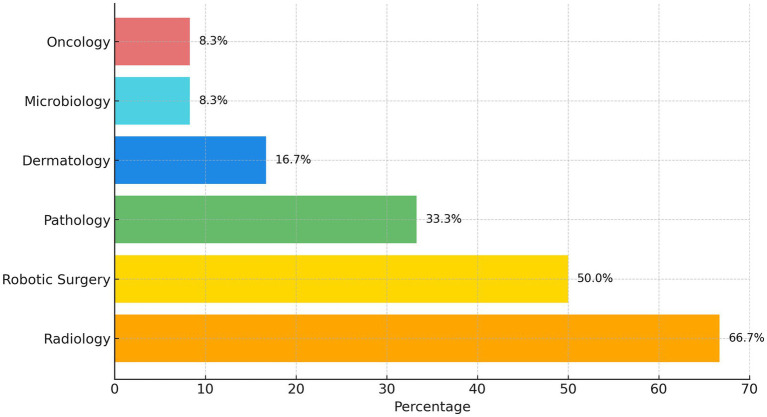
The most frequently cited healthcare specialties using AI.

Participants were asked in which application fields AI was used most frequently, in order to gain an overview of its perceived overall deployment. The healthcare specialties most linked to AI use are presented in Figure 1.

As illustrated in Figure 1, the specialties most frequently mentioned, in order of frequency, were Radiology (66.7%), Robot-assisted Surgery (50%), Pathology (33.3%), Dermatology (16.7%), Microbiology (8.3%) and Oncology (8.3%).

**Figure 2:**
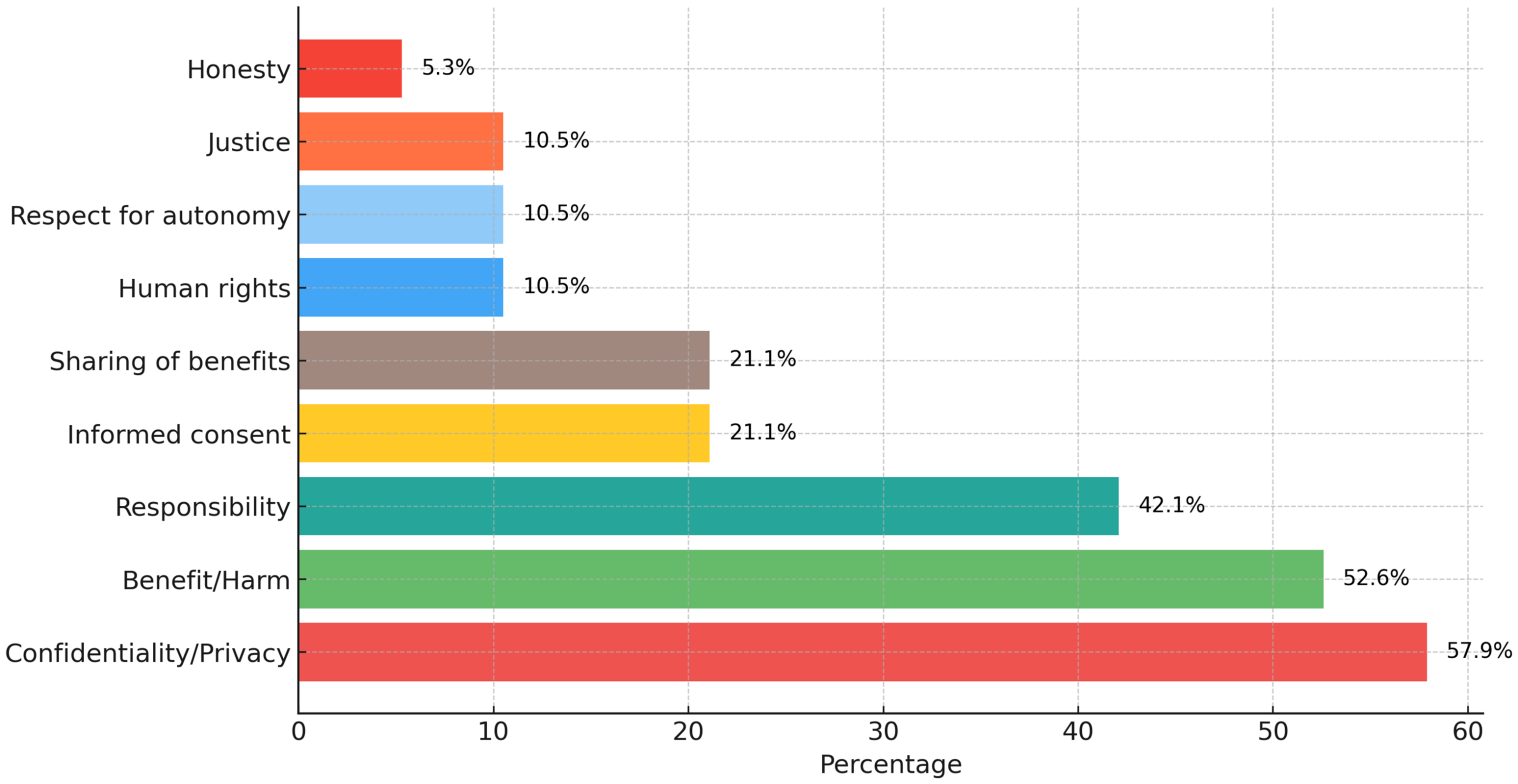
Bioethics principles related to AIIM.

One of the central aims of the current study was to identify ethical issues and principles related to AIIM. Content analysis was employed to determine the ethical issues mentioned in the interviews. An open-coded map was created based on previous research on AIIM (29–32). This then functioned as a reference for the closed coding. Figure 2 outlines the ethical principles involved in AIIM in order of frequency of reference.

As Figure 2 shows, the ethical principles most related to AIIM are, in order of frequency: *Privacy/confidentiality* (57.9%), *Benefit/harm* (52.6%), *Responsibility* (42.1%), *Informed consent* (21.1%), *Sharing of benefits* (21.1%), *Human rights* (10.5%), *Respect for autonomy* (10.5%), *Justice* (10.5%) and *Honesty* (5.3%). Quotes illustrating each ethical principle are listed in Table 2. For each quote, information about the participant involved is provided, namely their specialty and the length of time they worked as a specialist.

**Table 2:** Ethical principles and corresponding quotes.

Confidentiality/Privacy
*“[D]ata has become a very valuable product to use in advertising these days. It just occurs to me that patient data must be protected in such a way that companies or firms cannot use them in their advertisements and confidentiality is preserved” (Ophthalmology, 10+)* *“[P]atient information and confidentiality must be preserved. It should never be shared just because some state institution asked for it. Even now it is being shared, which is unethical.” (Radiology, 10+)* *From my viewpoint, this might be the most significant problem, for example personal data being made available, being aggregated and ending up in a format other people can see. Suppose I am a patient with AIDS and I do not wish anyone to know about it. However, everyone will find out that I have AIDS. Wherever I go this issue will confront me…so sharing data in this way and breaching confidentiality means serious potential harm” (Chest Diseases, 10+)*
Benefit/Harm
*“If you just go along trusting in the device and say on the basis of a rate supplied by the machine that the patient is not unwell, you will be prone to extremely serious problems. It may lead to a court case…when the judge asks why you ignored these patient parameters, why you did not arrange follow-up, why you relied on the machine, on what basis you relied on the machine, what the evidence shows and why the rates were low… what is our aim when we swear the Hippocratic oath? It is the health of the person and first ‘do no harm’…(Ophthalmology, 10+).* *“Profitability, effectiveness and profitability are extremely important, but human beings are their basis. Whatever you do to be effective and profitable, you do for a human being. If your nurse, staff, doctor or technician, nobody is happy any more… be as efficient as you like, the job will not get done.” (Radiology, 10+)*
Responsibility
*“Suppose we gave the information, obtained consent from the patient and at the end the patient was harmed. Who will bear responsibility…? Will it be the healthcare organization…or the doctor… or the firm that sold the application, or the software company that produced the application?” (Radiology 30+)* *“In the end the final decision lies with the physician and it is the physician’s responsibility. How he or she uses the prompts and algorithms that make up artificial intelligence is also a product of his or her knowledge, skill and decision-making. The person responsible is him or her…” (General Practice, 20+)*
Informed Consent
*“Ultimately we obtain consent from the patient for every type of procedure and, in my opinion, it is sufficient to solve the issues if there is a sentence stating that artificial intelligence forms part of the procedure or service you are receiving and the person provides consent about that subject.” (Surgery, 10+)* *“Currently one of our biggest handicaps is getting the patients to sign consent forms. We inform the patient every time we apply a treatment or are going to give a drug…At the moment there is no rule about whether to tell the patient or not when we are making use of an AI application or are applying a treatment or conducting a study in that way. However, that information needs to be given” (Radiology 30+)*
Sharing of Benefits
*“Actually, if you can deal with 100 patients in three days under normal conditions, with artificial intelligence, we will get to the situation where you can deal with three million patients. This creates a beneficial effect from a public health perspective. And we need to create a solution for the health problems of an individual living in the middle of nowhere. Under normal circumstances, you cannot sort anything out by sending healthcare staff there or by bringing that individual into the city center, but you can solve this issue with a very simple application.” (Surgery, 10+)* *“There are some locations where people and technology cannot reach… So, if these can be located in such a way as to support the existing human infrastructure rather than making up for a lack of human beings in these locations, it will deal with the deficiencies there and solve a lot of geographical issues.” (Radiology, 10+)*
Human Rights & Dignity
*“…already there exist established human rights … defined by the United Nations. Whatever else we say, we must abide by these rights…There must be nothing that restricts, directs or manipulates these rights.” (Medical Genetics, 20+)* *“Confidentiality definitely needs to be kept at the forefront…[W]hen we gather human data, we must do so by anonymization, not labelling, so that data cannot be matched to an individual. Consequently, respect must be shown for personal rights. Artificial intelligence may force us in this subject, in data collection. In short, there must be no identifier used. That is the key.” (Ophthalmology 10+)*
Justice/Fairness/Equality
*“If you utilize it to widen the provision of healthcare, this could genuinely be of benefit to the public. But if you just make some machine using artificial intelligence that can only be accessed by paying to a private hospital, then the benefit reaches no-one. It only serves those who have money. So, there is a difference between the two situations. Who will access it and how?” (Pediatrics, 10+)*
Integrity/Honesty
*“If the system is introduced to a patient as one that never makes mistakes, then ethical problems will begin…it must be stated at the beginning that these systems have a margin of error, even if it is very small. Patients should sign their consent on that basis. Otherwise ethical problems will arise.” (Surgery, 5+)*

Figure 2 shows that the highest perceived risk is related to Confidentiality and Privacy. Benefit and Harm must also be balanced. Responsibility and Autonomy are respected through the proper use of Informed Consent. Justice and Fairness, and Sharing of Benefit, are related to the respect for Human Rights and Dignity.

### Results of the thematic analysis

The main themes related to AIIM, derived from the basic problems identified, were: (i) Advantages (ii) Risks (iii) Limitations (iv) Precautions/Regulations and Policies. The related sub-themes are presented in this section and supported by examples from the interviews.

#### Advantages of AIIM

The main theme, “Advantages,” comprises four subthemes: “error reduction,” “increased speed,” “workload reduction” and “usability in training.”

The first subtheme of the advantages frequently mentioned by physicians was a reduction in errors, especially within diagnosis. The vast majority of participants stated that AIIM would greatly decrease errors or even eliminate them while improving diagnostic accuracy. Seen from this angle, AIIM appears beneficial.

*“…so, it provides a result more quickly and makes fewer mistakes than an expert. Naturally, this is something, depending on the physicians’ position, that they will prefer.” (Microbiology, 10+).*

*“We will greatly benefit, I think, from artificial intelligence since it will be able to warn us about points that we may overlook or forget to take into account because of a very heavy workload, and it will further increase our diagnostic accuracy.” (Ophthalmology, 10+).*

*“When taking a patient’s history, we may overlook important details. For instance, the patient may have a penicillin allergy and I might not know that because I did not ask about it, the patient may not have told me, and I could have prescribed medication from the penicillin group. This can create a serious legal situation and may seriously harm the patient. This type of application may make it possible to prevent this type of situation.” (Chest diseases, 10+).*

*“The reason is that [AI] will instantly eliminate human error and ensure standardization. Therefore, it may be considered as having a positive effect on patients.” (Radiology, 30+).*

The second sub-theme of the advantages relates to increased speed. The vast majority of participants cited faster service delivery as a key selling point for AIIM. A further, related advantage of speedier delivery was an increase in the time available to communicate with patients.

*“Therefore, since it will definitely shorten the work, for example by filling in a form before the patient enters hospital, if there is some data in front of the physician about the possible symptoms for that current complaint, [the physician can determine] potential diagnoses and what needs to be done before the patient arrives, and so with such a system in place these ten minutes can be used more effectively.” (Chest Diseases, 10+).*

*“The AI applications on pre-diagnosis, i.e., early diagnosis can increase the survival rates and shorten the time. People’s time is precious, physicians’ time is precious. It may really shorten the time needed for these things, especially certain investigations. Thus, as I see it, the clinician can spend more time explaining and communicating.” (Pediatrics, 10+).*

Most participants also felt AIIM enabled more precise diagnosis and freed up time for other tasks. Thus, AIIM would be both helpful and beneficial for clinicians.

The third sub-theme of the advantages was a reduction in workload. AIIM was seen as allowing physicians to avoid being overburdened, and enabling them to spend time on issues of real importance. However, this reduction in physicians’ burden could also eventually cause unemployment. The issue of unemployment is discussed in more depth under the section “precautions.”

*“It provides great advantages and reduces the workload. So, rather than looking for a lesion…when the system pre-scans it on my behalf, I can focus on what these lesions are and how they progress.” (Radiology, 10+).*

*“Once it starts to be used, it will make the work more straightforward…and release overburdened staff. However, the relief of this burden will later create further problems for physicians when jobs become limited.” (Radiology, 30+).*

The fourth subtheme of advantages was the use of AIIM in education. Many participants stressed the educational value of AIIM for physicians and students, alongside its other advantages.

*“As a helping tool for physicians, its use in the training of physicians must be supported.” (Dermatology, 10+).*

*“I think it would be better if we made more use of AI in teaching students. For instance, an ophthalmologist who has not yet reached the level of specialist, or a junior assistant, may not notice something very small hiding on a background of diabetic retinopathy. Therefore, if the AI says that there is a 99% probability of a lesion…and it can also say at what stage the lesion is, that would be very good for student education. That is why it can be used in student education.” (Ophthalmology, 10+).*

#### Risks of AIIM

Risks and negative aspects of AIIM were frequently mentioned alongside its advantages. The risks mentioned were of two types: those related to clinicians and those concerning reliability.

The *risks for physicians* encompassed potential unemployment, the need for restructuring, and human devaluation. The following excerpts reveal the concerns that physicians have of becoming redundant due to AIIM, the need for restructuring and planning to prevent this outcome, and the fear that human beings may lose their sense of worth. As the use of AIIM spreads, respondents feared physicians’ roles would be taken over by robots and software. Thus, the medical workforce would become insignificant.

*“If AI takes over some duties from physicians, the number of physicians will probably decrease. A job that five people can do can be done by one person. Or a robot… For example, instead of radiology taking a film, it is the device that does it, and it transfers the appearances and can tell you the diagnosis 95% of the time. [AI] is entering into a phase where it will eventually end many people’s jobs, if you look closely enough.” (Surgery, 5+).*

*“Are you going to close the medical schools now? There are lots of things like that. So, it is necessary to reduce the quotas for medical students, but I guess it is also necessary to set up medical informatics undergraduate programs to replace the ones taken away. Who will do what I am talking about? Someone has to do the programming.” (Medical Genetics, 20+).*

*“There is a prediction that the human factor will become less valuable wherever artificial intelligence starts being used. I have no clear idea. Is artificial intelligence used in industry? Yes, it is. This of course causes people to become more and more excluded, and worst of all, it makes people feel valueless. This is a bad thing, human beings feeling worthless.” (Surgery, 20+).*

The other type of risk for AIIM concerned reliability. *The risk of unreliability* was felt to be related to an undue focus on profit, a degree of insensitivity to individual differences, and faulty data entry. The participants stressed concern about malicious software and linked it to an over-emphasis on profit. Unreliability could also arise by ignoring patients’ differences, leading to the risk of atypical patients (e.g., ethnic minorities) being disadvantaged. If algorithms lack evidential support, they may also be unreliable, participants warned.

*“AI will provide the diagnosis and write the prescription. When it writes the prescription, does that mean the pharma companies will become involved? Are the drug companies going to interfere? To do that, maybe malicious software will be installed and whoever on earth is controlling this AI system, if the pharma companies make an agreement with them, they will become these firms’ puppets.” (Radiology, 30+).*

*“It seems to me that detecting changes according to the patient’s skin, and so on, is no easy task. Since a lesion on the arm of a slender individual is not the same as a wound on the arm of an overweight and hairy person, it will not be able to diagnose very well, since it will not detect the criteria it is looking for.” (Microbiology, 10+).*

*“While the algorithm is being constructed, the evidence-based data must be uploaded to devices. The number of diseases that can be encompassed by this evidence-based data is fundamentally low…. In such cases, what the devices will do and which algorithms will be uploaded, whether it is one algorithm in this hospital, while another hospital works with another algorithm, or whether it is the same device, the same machine…? These matters will be very contentious.” (Radiology, 10+).*

#### Limitations on using AIIM

The limitations reveal the physicians’ negative views toward an expansion of the use of AIIM. The limitations comprise three subthemes: physician-patient relationships; decision-making; and the application of the technology. AIIM could harm the *physician-patient relationship* and decrease trust (33). In normal circumstances, this human relationship is therapeutic in itself. AIIM may also lack a holistic view of the patient to inform its decisions. For these reasons, patients’ trust may be lost and AIIM would be of limited benefit.

*“After all, we all know that, in the recovery of a sick individual, the contact with a physician, the feedback patients get from the physician, and the words the physician uses toward them, their approach and sometimes even their touch produce 50% of the effect…. These effects, this sense of compassion, are very effective, especially for people who do not have a very grave illness.” (Medical Microbiology, 10+).*

*“The final diagnosis, of course, goes together with the physicians’ own clinical decision processes and with the holistic evaluation of the patient…when further investigations are required, the results are considered alongside some other clues…to make a decision about a patient’s particular situation and to discuss it with them…to say this, explain, deliver bad news, for example…. I cannot even imagine how a robot could deliver bad news. It’s impossible.” (General Practice, 20+).*

*“It seems to me that we should be able to put ourselves in the patient’s place, we should touch the patient’s head, we should establish a relationship with them not just based on their disease but also on their humanity. In our time, our teachers would tell us that 60 to 70% of the efficacy of treatment comes from trust in the physician. That trust comes about through human emotions. Therefore, if I think about whether AI should be introduced into medicine or not, I believe it should not.” (Surgery, 35+).*

The remaining sub-themes of the limitations relate to decision-making and the scope of AI application. Participants overwhelmingly felt that physicians should supervise AIIM when it comes to decision-making, and that the final responsibility should lie with a human being, not a machine. Decisions made by diagnostic AI were easier to accept than those used in surgery.

*“The final decision must be a human one. After all, since we always make decisions with our patients, artificial intelligence will not be able to make decisions for a patient, just as a physician cannot decide on behalf of their patient. That communication should be between two human beings. I do not want to tell a robot about my problems. I would not want, as a patient, to tell a robot why I accept or do not accept a particular treatment. It must not be like that.” (General Practice, 25+).*

*“Another thing, of course, is that at first it might seem nice and easy to do all the work remotely, but after a time when there is no human to interact with, it will leave patients in an unsafe situation. I mean, sometimes a physician is a shoulder to cry on. Do you see what I mean? These tasks will not be easy since the system cannot replace this.” (Surgery, 15+).*

*“[T]he decision made by a machine, which is deprived of emotions, will not always be the same as a decision made by a physician who can empathize with another person. Therefore, although these technological solutions maintain a high rate of accuracy, as I said, the final decision must be made by the physician in the cases that will affect a human life in a major way, such as a decision to undergo surgery, or in the case of an organ transplant.” (Microbiology, 10+).*

*“The field in which AIIM can most easily be applied is in diagnosis. It does not touch the patient at all…. The biggest challenge is to apply AI in the field of surgery…” (Surgery, 10+).*

### Precautions, regulations and policies about AIIM

The final theme, “precautions, regulations and policies,” comprises the sub-themes of “education,” “accreditation” and “data security legislation.” The need for both healthcare professionals and society at large to know about AIIM was stressed. AI systems should be accredited and regulated by scientific societies so as to be open to being audited. This role should be undertaken by impartial, supranational parties. Legislation-protecting data would be challenging to draft, given the current uncertainties about limitations and legal obligations.

*“Probably one of the most important aspects is education. The public, people, society, especially the public, I reckon, as well as the most critical part of society, healthcare workers, need clear information about AI.” (Medical Education, 10+).*

*“The use of smart systems will, of course, be controlled and regulated…. They must be accredited…and they need to be accepted by the scientific community. Even smart health records…. [T]here should only be one or a few in practical use and those that do exist should be certified.” (General Practice, 20+).*

*“There has to be a truly impartial international platform for this…audits should also be open to all developers.” (Radiology, 30+).*

In summary, the pooled thematic analysis showed that physicians see advantages in AIIM, namely the increase in work speed, the reduction of errors, the reduction of workload, the improvement of service quality, and the improvement in training. The risks identified were increased unemployment, a devaluation of humans, a focus on profit, and faulty data. Limitations arose from harming physician-patient relationships and the need for supervised decision-making. Precautions envisaged included AIIM training for both professionals and the public, international audit, and the development of global standards.

## Discussion

This study aims to detect the prominent ethical principles and dilemmas that concern the application of Artificial Intelligence in Medicine (AIIM), and it attempts to understand the viewpoints of physicians from various fields of expertise on the use of AI-powered systems in their professional lives. As mentioned above, AIIM covers many areas in medical practice and clinical decision-making such as diagnosis, treatment, disease prediction, patient management, administrative applications, and electronic records (34), and it provides numerous benefits as well as present ethical challenges concerning medical practice and healthcare provision (35).

### Ethical principles and decision-making

From an ethical standpoint, investigations of technologies that appear beneficial need to ask “who benefits?” as well as “to whom is it a benefit?” The underlying values need to be questioned in order to come up with a value-laden argumentation to reach an ethically satisfactory conclusion. Siau and Wang (16) argued that since AIIM behaves in specific ways, we can apply ethical reasoning to evaluate its decisions and actions. This reasoning led Jobin et al. (36) to consider transparency, justice, fairness, non-maleficence, responsibility, and privacy as the key concepts for an ethical use of AIIM. By scrutinizing the issue from AI-based decision support systems, Braun et al. (37) drew attention to the transformations of modes of interaction in the clinic among clinicians, patients, and machine; they questioned this fact in terms of normativity challenges, i.e., trustworthiness, transparency, agency, and responsibility, and consequently supported human control over AI-based decision-making processes to ensure professional competency and patient beneficence (37).

Our study contains not only similarities with the recommendations and findings in the literature, but also reflects the personal experiences of health professionals in their clinical practice. The results of this research indicate that participants considered confidentiality and privacy as the ethical values that are most at risk. They emphasized the need to balance benefit with maleficence, and to restrict the risks by prioritizing patient welfare. They attached importance to a responsible use of AI by openly stating that the final decision should rest with individuals, that autonomy should be weighed with responsibility by clearly defining the duties of the parties, and by physicians taking responsibility for obtaining the patients’ informed consent. Participants cautioned against the harmful effects and misuse of this technology, and insisted that AI-powered systems should operate with the goal of achieving justice, fairness, and a sharing of benefits. Physicians put emphasis on universal, valid, auditable, honest, human rights-based use of AI systems, to be governed by international law and supra-national ethical guidelines. The physicians interviewed demanded AIIM that offered universal benefit, exhibited transparency, and respected human rights.

Participants in our study viewed AIIM as advantageous because it reduced the risk of error, allowed them to act more quickly, especially in diagnosis, lightened their workload, and assisted them in medical education. AIIM was particularly suited for analyzing visual data in radiology, histopathology, and retinal photography. Further benefits of AIIM were a better image quality and a more organized storage of data. These findings resemble the argumentation by Mintz and Brodie (18) that AI embedded in electronic records enables the possibility to calculate disease risk and achieve early diagnosis. On the other hand, Mittelstadt et al. (38) rightly pointed out that the inscrutability of the evidence used by AI decision-making algorithms lead to opacity, that misguided evidence leads to bias, unfair outcomes lead to discrimination, transformative effects lead to challenges for autonomy and informational privacy, and traceability leads to moral responsibility so as to guide ongoing and future studies. Thus, Kempt and Nagel’s (39) elaborate ethical and epistemological challenges of using AI in clinical diagnostic contexts in view of attributing the responsibility between health provider and machine have been repeatedly and critically stated by the physicians interviewed in our study. Therefore, as argued by Kempt and Nagel (39) and as we also strongly emphasized in our study, the primacy and precedence of the physician throughout the clinical decision-making processes is of the utmost importance, and also supports the role of AIIM as a secondary opinion source to safeguard accuracy and explainability, and to resolve disagreements. As a matter of fact, Grote and Berens (40) investigated the opportunities and pitfalls of algorithmic decision-making in healthcare, such as the use of machine learning, with respect to paternalism, moral responsibility, and fairness. This challenge justifies the emphasis we put on keeping a balance between autonomy and responsibility in order to enhance clinical reasoning, raise patient beneficence, and reduce paternalism by utilizing AIIM as an asset.

Our study identified two types of AIIM risks: one affected physicians, the other concerned reliability. The risks to physicians were unemployment and being devalued as a human being. These fears may underlie the negative bias some professionals have toward AIIM. The way patient data could be shared with commercial developers was considered a reliability risk. Skewed or incomplete data would mean AIIM is insensitive to individual differences and would result in systems that are more beneficial to a particular gender or race.

Since AI may reduce the need for human workers, there are concerns it may cause unemployment. At present, however, it seems that in healthcare, AI plays an assistive role, and it does not seem poised to replace physicians, at least for the moment (26). Our study showed that physicians perceive a focus on profit as a source of potential unreliability. The literature highlighted a series of risks related to secure and confidential data transfer and agreed that a unilateral focus on profit would indeed present several risks (41). Breaches of security may impact individuals on a large scale, hence models need to be created that will allow individual rights to be respected, data to be stored in a highly secure way, and a regulatory framework to be built to ensure these outcomes (42). According to the results of our study, how far one risk prevails over the others depends on where the priority is placed. This issue will also be tackled in the “Precautions” section below. Nevertheless, the unreliability of data, which is one of the subthemes, poses another major risk that may lead to insensitivity to individual differences. As argued by Currie and Rohren (43), the under-representation of socioeconomic, cultural, or ethnic groups in insufficient datasets creates bias (43, 44). This issue is also connected to the precautions we recommend in our study (see “precautions” section below). In the literature, specific examples of overgeneralization were noticed for the Framingham Heart Study, a cohort study of cardiovascular health. These data concerned exclusively white individuals and led to inaccuracies when applied to Black people (41). Datasets can be rendered more representative by including under-represented groups and, thus modified, can lead to more accurate results (45). Inequality is a concern when minorities receive inferior service or cannot even access healthcare (43). Our study findings indicate that equality may not be attained when AIIM is insensitive to individual differences, an issue grouped under the “reliability risks.” In our findings, the risks related to reliability can be compared to the “black box” concept, a key issue about reliability, accountability, and transparency, often tackled in the literature (46, 47). This issue was alluded to by respondents when discussing the reliability of AIIM. Even where the input, output and algorithm used are explicit, AI may arrive at decisions that seem mysterious to a human being (48). Participants questioned how liability could be correctly apportioned in the case of harm caused by AI—is the clinician, the hospital, or the AI developer responsible? The black box nature of AI can create an ethical and legal conundrum (49). As rightly cautioned by the participants of our study, the black box issue in AIIM puts patient-centered medicine at risk regarding transparency (50), accountability, and explainability (51).

As AI in healthcare expands, transparency and trust become crucial. Explainable Artificial Intelligence (XAI) helps make AI decisions clear and understandable for clinicians, ensuring accurate results with justifiable outputs (42). XAI maintains healthcare professionals’ trust by explaining AI-driven recommendations, enabling informed decisions. It also addresses ethical concerns like bias and fairness, ensuring AIIM systems are equitable and reliable. Thus, integrating XAI into AIIM enhances transparency and supports the ethical safeguarding of patient welfare and dignity (39). Future AIIM development should prioritize XAI to build trust and accountability.

### Limitations

The areas where AI was seen as most likely to be adopted were radiology and robot-assisted surgery. In both fields, AI assists clinicians by simplifying their tasks. In our study, a key limitation that would arise from the use of AIIM was the damage done to the physician-patient relationship. AI was equated by some participants to an emotionless robot, unable to communicate meaningfully with a human. Participants conceded that AIIM would be fast and generally error-free, but the vast majority of respondents felt that human control over decision-making should not be relinquished. Even when it comes to the diagnostic decision process, where AI appeared most reliably applicable, concerns remained about letting AI be entirely in charge of the final decision. There is a need for future detailed studies of the points of view of physicians when faced with deciding whether to accept the conclusions reached by AI.

The issue of responsibility plays a pivotal role in the risks and limitations of AIIM. The black box problem complicates the whole issue. There is also the issue of bias: skewed data will lead to skewed decisions. While bias may originally be accidental, the systematic under-representation of minorities may lead to it becoming systemic. According to our participants, physicians risk reinforcing bias by supporting conclusions that match their prejudices. As nicely put by Kiener (47) if AI is not simply an aid but the decision-maker, serious problems may occur. Thus, although not explicitly stated during our study, the participants tended to agree the view in line with symbiotic AI in AIIM that refers to a collaborative partnership between AI systems and human professionals, enhancing each other’s strengths for better patient care (17). This approach combines AI’s data processing power with clinicians’ judgment and empathy, ensuring adaptable and responsive healthcare solutions by which it fosters continuous interaction, improving accuracy and building trust by positioning AI as an extension of human expertise rather than a replacement (42). This method also addresses ethical concerns by ensuring human oversight in critical decisions.

### Precautions

The key precautions stressed in our study were education and audit. It is a necessity to train not only physicians and other health professionals but also all stakeholders in the use of AI. Participants considered the existing national legal and constitutional safeguards for data inadequate to circumscribe the risks, and a wish for global audit and standardization was strongly voiced. Participants also emphasized that legal policies that fully consider data security should be prepared before the use of AI becomes widespread in the healthcare context.

As AIIM proliferates, healthcare professionals will be required to be informed about its development and how it should be used in their practice. For this reason, a limited understanding of AIIM, the negative attitudes of clinicians, and a fear of unreal risks, can all lead professionals to underuse AI technologies in health services (52). Charow et al. (53) found existing professional education on AIIM to be too limited. They noted that the existing training focused exclusively on AI development, whereas training on where and how to use AI to aid decisions needed to be further developed. Our study findings agreed with the conclusions reached in the literature by advocating the need for detailed training but went a step further by also recommending that such training be extended to all stakeholders, not just healthcare professionals.

Another key ethical issue is transparency. The use and development of AIIM must be transparent to ensure the protection of all stakeholders. AI decisions should be comprehensible and need to be explainable should the need arise. Our study emphasized the implementation of audit as a precaution. While regulations already exist, such as the EU General Data Protection Regulation, new standards are needed to allow the transparent audit of AIIM systems. The P7001 Standard of the Institute of Electrical and Electronics Engineers (IEEE), “Transparency of Autonomous Systems,” has been developed with this goal in mind (54).

In their comprehensive analysis, Floridi et al. (52) delved into the benchmarks of a Good AI-Society and investigated opportunities, risks, principles and recommendations connected to it, on the basis of the bioethics principles of beneficence, non-maleficence, autonomy, justice, and explicability. They maintained that the use of AI could enable human self-realization, enhance human agency, increase societal capabilities, cultivate social cohesion to achieve not only good medical practice bolstered with AI-powered tools, but also a more democratic society at large (52). Thus, this could be linked to an approach known as Human-in-the-Loop (HITL) in AIIM which ensures that AI systems are used as tools to support, not replace, human decision-making in healthcare. HITL allows clinicians to review and override AI recommendations, preserving ethical standards and patient-centered care (55). This approach mitigates risks by enabling continuous human oversight, crucial for maintaining accuracy and addressing complex medical decisions. Prioritizing HITL methodologies in AIIM reinforces the essential role of healthcare professionals, ensuring AI complements their expertise.

The physicians who took part in our study attached importance to the need of creating an international regulatory framework that would function in line with human rights and dignity, as well as with an ethical code of conduct. This implies that the supra-national recommendations and statements pronounced by the international organizations to which Turkey is affiliated are apt to respond to this significant need.

### Study limitations

Finally, some limitations of our research need to be acknowledged. Even though the physicians in our study worked in various fields of expertise, they all came from a single private institution in Turkey. Since qualitative research does not aim to generalize but should represent as many different views as possible, it could be objected that our findings do not accurately represent the range of views held by employees in different cultures and institutions (for example, physicians in state institutions with more limited resources) (28). Furthermore, since AIIM is not yet widespread, the opinions expressed are not specific to particular implementations (56). Future studies will need to focus on specific AI applications already deployed or under development. Additionally, future studies will have the benefit of using qualitative findings to develop questions that target more specific areas within clinicians’ attitudes to AIIM.

## Conclusion and recommendations

Understanding attitudes toward AIIM is important to gain a comprehensive perspective on the potential consequences of such technologies becoming more widespread in the future. Our study’s fundamental aim was to investigate the experience and thoughts of physicians about the use of AIIM in their field, and to set out the ethical and social issues physicians see as relevant. When the findings are reviewed as a whole, we observe an emphasis on the dilemmas AIIM entails. Alongside the advantages, there are many limitations and risks. The study reveals a perceived need for precautions to be embedded in healthcare policies to counter the risks discussed. These precautions need to be multi-dimensional.

In the light of our findings, it is evident that physicians trust AI to help them reach a more efficient and effective and acknowledge AI will be beneficial by saving time. However, they also consider that the physician’s relationship with the patient has a positive effect on treatment and doubt this effect can be replicated by AI systems. Furthermore, there are concerns about privacy and the protection of confidential personal data, and fears of potential data misuse. Respondents also emphasized the need to prioritize patient benefit. Autonomy and responsibility could be respected by granting physicians priority over AI in decision-making. To prevent any harmful use of AIIM, this principle needs to be adhered to. For AIIM to be well-integrated and accepted, the participants stated that any system should ensure fairness and equality, as well as the sharing of benefits. It should reach out to excluded and marginalized groups. AIIM must operate with transparency, accountability, and auditability.

One of the most challenging ethical problems relates to how patients from different socioeconomic status could access AIIM given its high research and development costs compared to more traditional approaches. Conversely, diagnostic AIIM could facilitate access to healthcare for underprivileged groups by reducing the cost of diagnosis.

In conclusion, the physicians’ views on the ethical use of AI can be used as a basis for the development of good practices in the field. AIIM built on revised ethical principles specifically adapted to AI can provide better healthcare systems. We should keep in mind Mittelstadt’s (57) emphasis that “principles alone cannot guarantee ethical AI” and further investigate the point of views and experiences of the concerned agents; this, in turn, can foster accuracy and precision in medical practice and reduce the workload by assisting physicians during clinical tasks. AIIM that functions transparently and respects the public interest can be an inspiring scientific innovation for humanity. AIIM developed by putting at the forefront ethical principles of human dignity may preserve humans even from self-induced harm and transform healthcare in positive, beneficial ways.

## Data Availability

The data supporting the findings of this study, collected through semi-structured interviews, are available from the first author (Fatma.Kahraman@acibadem.edu.tr) upon reasonable request.
